# Supplementary figures and images for: Function of Anthocyanin and Chlorophyll Metabolic Pathways in the Floral Sepals Color Formation in Different Hydrangea Cultivars
Source: Plants (Basel). 2025 Feb 28;14(5):742. doi: 10.3390/plants14050742 (PMC11901515; doi:10.3390/plants14050742)

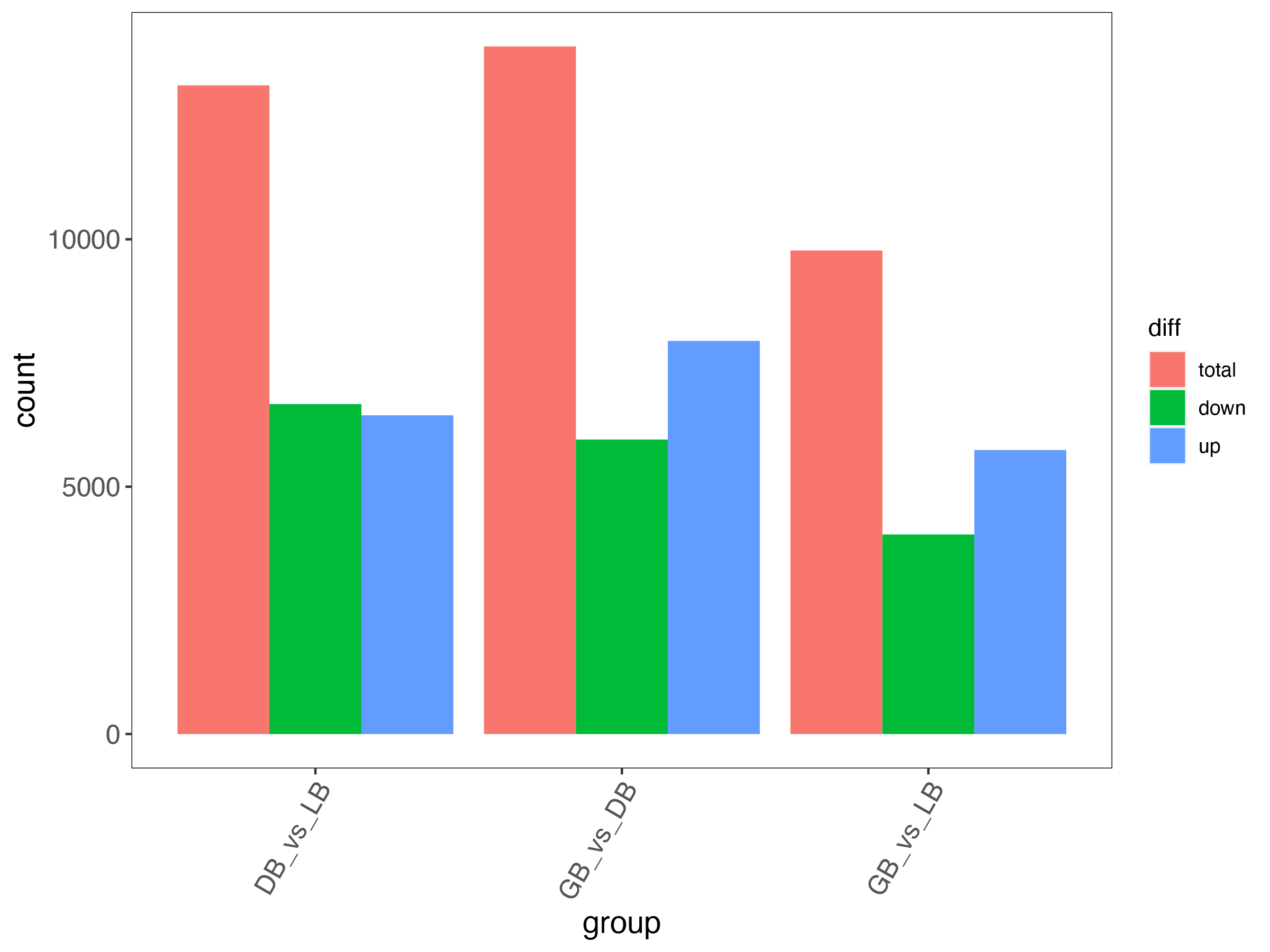

Supplement: Supplementary file 1 [file plants-14-00742-s001.zip › Supplementary Figure S3.jpg]

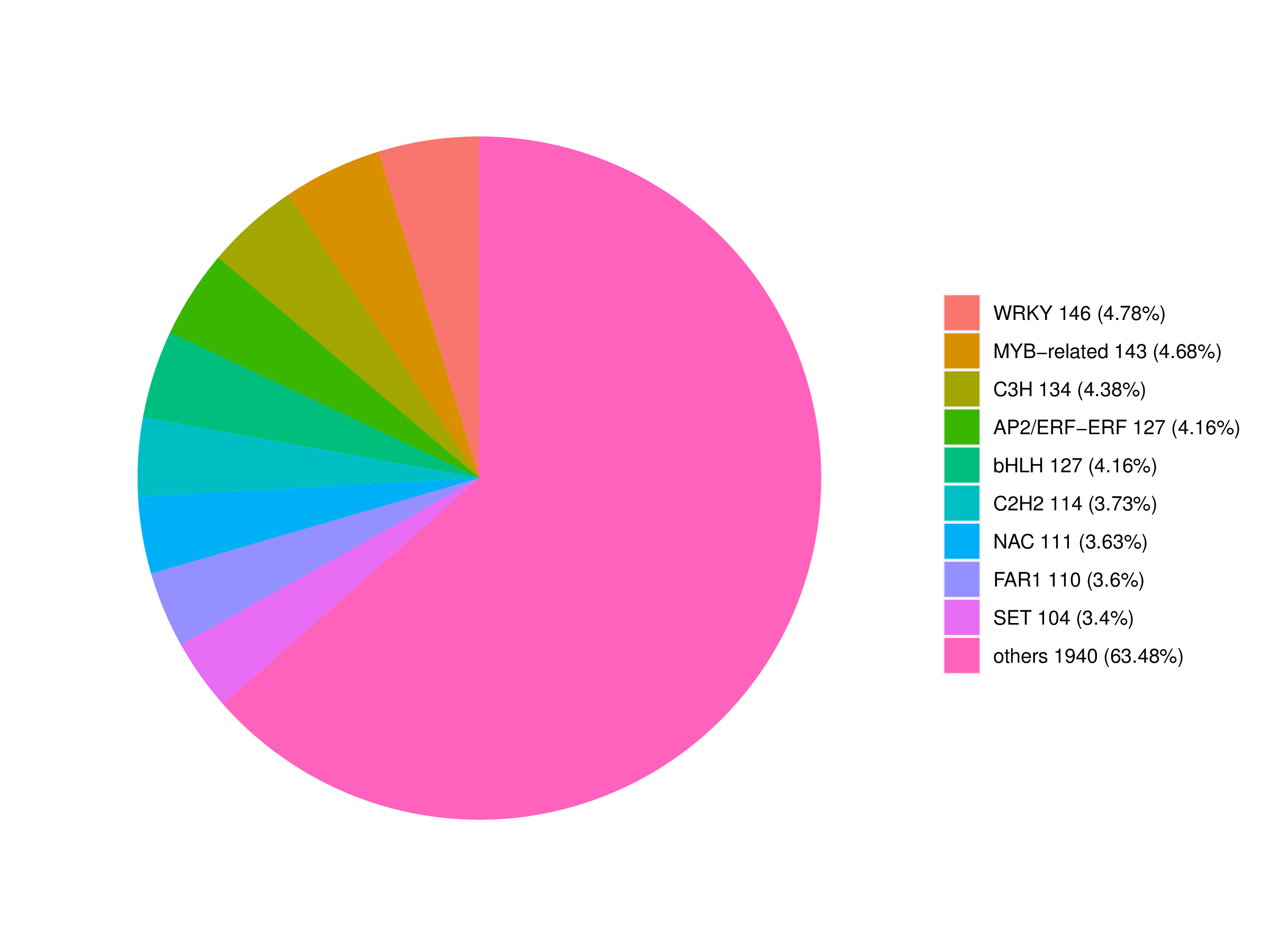

Supplement: Supplementary file 1 [file plants-14-00742-s001.zip › Supplementary Figure S4.png]

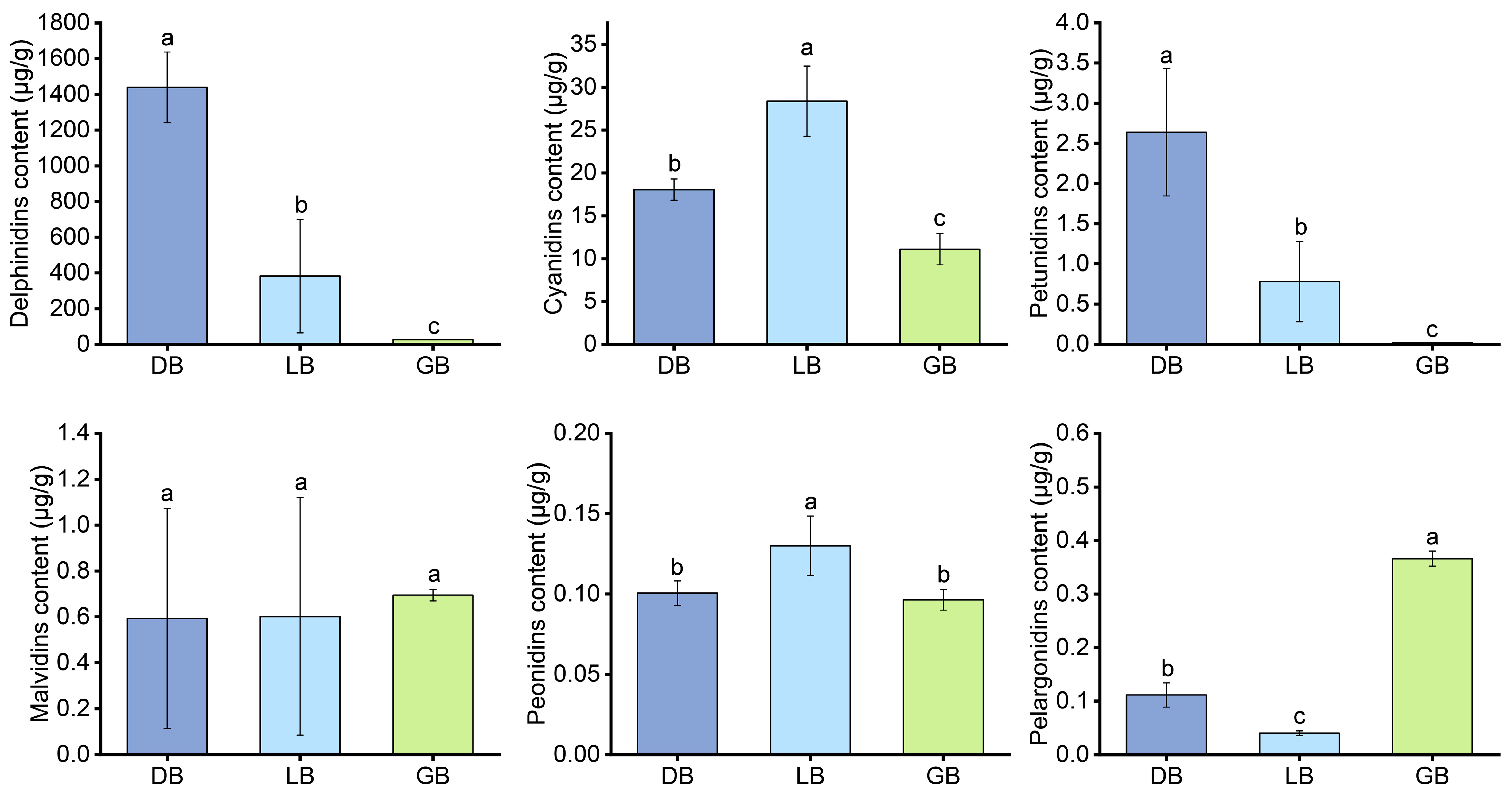

Supplement: Supplementary file 1 [file plants-14-00742-s001.zip › Supplementary Figure S1.jpg]

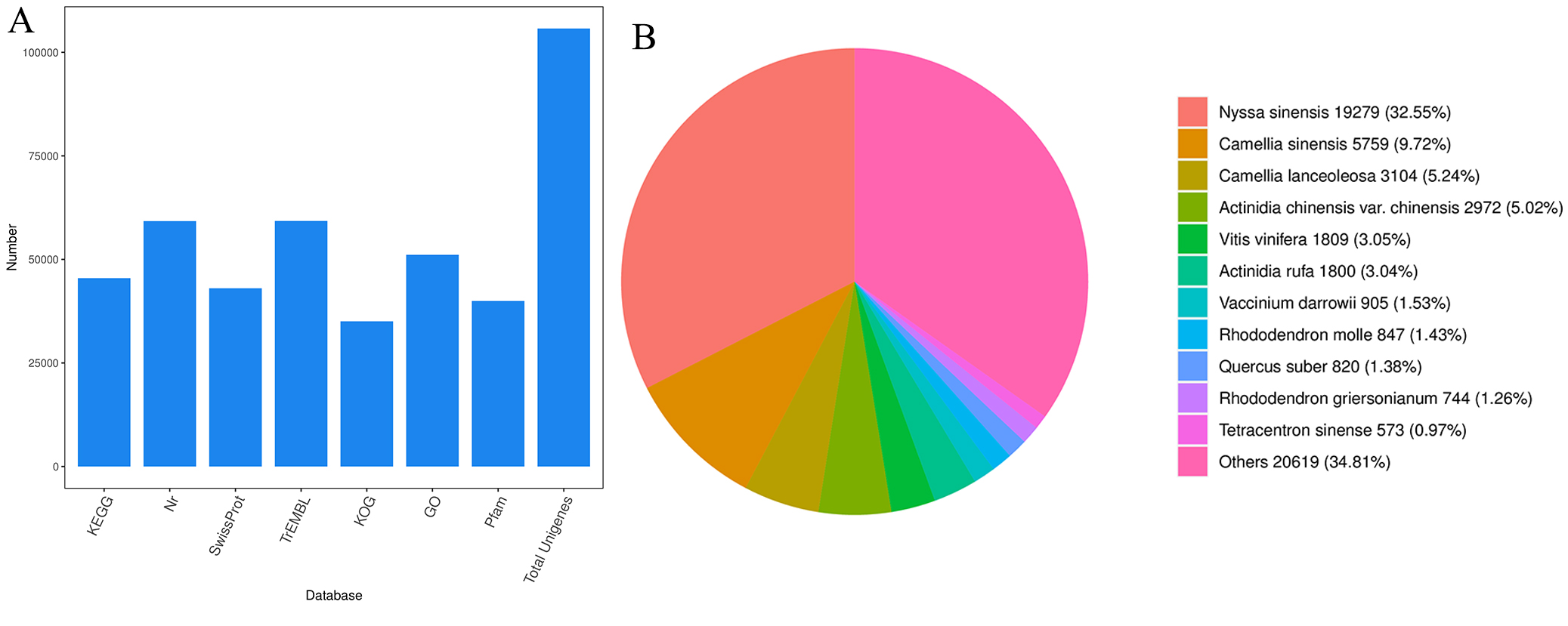

Supplement: Supplementary file 1 [file plants-14-00742-s001.zip › Supplementary Figure S2.jpg]
